# Resident physicians’ mental health during COVID-19: Advocating for supports during and post pandemic

**DOI:** 10.36834/cmej.70493

**Published:** 2020-12-07

**Authors:** Emma Gregory

**Affiliations:** 1 Department of Psychiatry and Behavioural Neurosciences, Michael G. DeGroote School of Medicine, McMaster University, Ontario, Canada.

## Abstract

There is already considerable evidence of how this novel corona virus (COVID-19) has had a major impact on our mental health and wellbeing. We are reminded of the mental health consequences of previous infectious disease outbreaks, not only for the public, but for frontline healthcare workers. Yet the lived experiences of resident physicians are missing from this discussion despite them being essential to the COVID-19 response and continuing to provide care during this time. The author asserts that considering what is known about the mental health effects of frontline healthcare work during previous outbreaks, residents are at risk given their role as physicians. In addition to baseline systemic stressors that put residents at risk of mental distress, they also face COVID-19 related stressors that exacerbate the risk given their role as trainees too. The author acknowledges and welcomes several rapid responses to residents’ developing mental health needs from medical leaders across Canadian hospitals, programs, and resident bodies. Ultimately, however, medical leaders need to advocate for and implement changes that will support residents’ mental health now and in the long-term well after COVID-19 has left its mark.

## Abstract (fr)

À partir d’une recherche sur l’actualité qui a émergé de la Chine et des autres pays qui ont été touchés les premiers par la maladie à coronavirus 2019 (COVID-19), il existait déjà des données relativement à son incidence majeure sur notre santé et notre bien-être. On nous rappelle les conséquences des épidémies antérieures de maladies infectieuses sur la santé mentale, non seulement pour le public, mais également pour les travailleurs de la santé de première ligne. Pourtant, les expériences vécues par des médecins résidents sont absentes de cette discussion, même si elles sont essentielles à la réponse au COVID-19 et à la continuité des soins offerts au cours de cette période. L’auteur affirme qu’en tenant compte de ce qui est connu des effets sur la santé mentale du travail de la santé de première ligne au cours des éclosions antérieures, les résidents sont à risque étant donné leur rôle de médecins. En plus des agents stressants systémiques réguliers qui mettent les résidents à risque de détresse mentale, ils sont également confrontés aux agents stressants liés à la COVID-19 qui aggravent également le risque alors qu’ils sont stagiaires. L’auteur reconnaît de nombreuses initiatives des dirigeants médicaux dans les hôpitaux, les programmes et les organismes de résidence au Canada qui sont accueillies comme des réponses rapides à l’évolution des besoins en matière de santé mentale des résidents. En dépit de ces actions positives l’auteur demande aux responsables médicaux de continuer à promouvoir et à mettre en œuvre des modifications au soutien en matière de santé mentale maintenant et au cours des années à venir, après la COVID-19.

## Introduction

From the moment when Coronavirus Disease 2019 (COVID-19) first emerged to the most recent months of global spread, it has been shaping our wellbeing in profound ways. Following previous infectious disease outbreaks, research showed how frontline healthcare workers experienced both short and long-term psychological distress. There is already research during COVID-19 that supports similar findings and recommends improved access to mental healthcare.^1^ In their roles as trainees and physicians, residents are participating in COVID-19 efforts, yet there are few articles that explore their experiences. Normally residents are affected by work-related stressors including lack of control, unpredictable case load, stressful work situations, modest financial remuneration, and work-life conflicts.^2^ Indeed, in a 2018 Canadian Medical Association survey residents were more likely to report symptoms of burnout, depression, and suicidal ideation than staff.^3^ During COVID-19 residents face a variety of disruptive changes to work and training that exacerbate the effects of regular work-related stressors and expose them to unprecedented ones.

## COVID-19 related stressors

### Increased work

Many residents are experiencing increased work as a result of covering peers and staff that may be off work due to being pregnant or immunocompromised, or those who have been exposed and are self-isolating or are ill.

### Cancelled leaves

Residents normally have vacation and professional leave, but many have had these cancelled because of high clinical needs. While some residents may rollover these days to the next academic year, this is not guaranteed. Nor does it address stress in the moment, when relief is needed.

### Redeployment

Given their junior status, residents are recruited to be redeployed to support emergency medicine, internal medicine, and the intensive care unit. This is mandated and there is much uncertainty because redeployment needs are fluid.

### Unsafe work

Unlike staff that have more clout to refuse unsafe work or to secure pandemic pay, such as in New York during COVID-19 when physicians could not access adequate personal protective equipment, ^4^ many residents cannot be so assertive because of power imbalances and fear (often well grounded in reality) of retribution.

### Training extensions

There is disruption to training if residents are redeployed in any capacity, if they are working from home, or are off work. Some residents may require training extensions that could delay their personal and professional goals.

### Certification/licensing

Residents applying to subspecialties or fellowships are impacted as well as final year residents who are experiencing delayed certification exams. For the latter, they are left with provisional licensing and supervised practice as they transition to staff.

### Evolving mental health needs

General experiences in the moment may include fear of being infected and transmitting to others, strains of social isolation, poor training/support, and variable access to personal protective equipment.^5^ Recognizing that stressors exist in addition to those shared by most frontline healthcare workers, it is critical that medical leaders respond. Based on resident discussion via forums and media during COVID-19 in Canada, some medical leaders are quite responsive to residents and others are less so. Residents are deserving of a timely and comprehensive response, which involves shaping work culture, environments, and services to protect and support them in these extraordinary times. Some initiatives to date include online hubs that collate information on a variety of wellness-related topics; wellness webinars and programming such as mindfulness; academic day wellness content that is related to COVID-19; program, University, and even national peer support groups; and, a move to virtual and extended-hours mental healthcare for residents in distress.

## Mental health support post COVID-19

Medical leaders, in the midst of this COVID-19 pandemic, recognize not only the vital role that residents have as frontline healthcare workers, but also their vulnerability. Normally residents are at risk of psychological distress such as burnout but with the added impact of COVID-19 they experience even greater degrees of stress. Given their junior position as trainees, they may not have the same power and avenues as staff do to advocate for themselves. It is therefore necessary that wellness champions, e.g. medical education leaders, recognize and respond to residents’ needs. Considering what is known about the mental health effects of frontline healthcare work during infectious disease outbreaks, as well as the ongoing work-related stressors for residents, medical education leaders should provide robust mental health supports for residents now and in the future. We need to prioritize resident wellbeing immediately and after the pandemic so that services are sustained. The mental health needs of residents are serious and will not disappear even when COVID-19 does.

## References

[1] Kang L, Ma S, Chen M, et al Impact on mental health and perceptions of psychological care among medical and nursing staff in Wuhan during the 2019 novel coronavirus disease outbreak: a cross-sectional study [published online ahead of print March 30, 2020]. Brain Behav Immun. 2020;pii: 10.1016/j.bbi.2020.03.028 PMC7118532 32240764

[2] Mian A, Kim D, Chen D, Ward WL Medical student and resident burnout: a review of causes, effects, and prevention. J Fam Med Dis Prev. 2018;4(4):1-8. 10.23937/2469-5793/1510094

[3] Canadian Medical Association. CMA national physician health survey: A national snapshot [Internet]. Ottawa: Canadian Medical Association; 2018 29 p. Available from: https://www.cma.ca/sites/default/files/2018-11/nph-survey-e.pdf [Accessed May 2020].

[4] Corley J Doctors in training are dying, and we are letting them down. Forbes Innovation [Internet]. 2020 Apr 5 Available from: https://www.forbes.com/sites/jacquelyncorley/2020/04/05/doctors-in-training-are-dying-and-we-are-letting-them-down/#2043e3a86cdc [Accessed May 2020].

[5] Tam CW, Pang EP, Lam LC, Chiu HF Severe acute respiratory syndrome (SARS) in Hong Kong in 2003: stress and psychological impact among frontline healthcare workers. Psychol Med. 2004;34(7):1197–1204. 10.1017/S0033291704002247 15697046

